# The role of body height as a co‐factor of excess weight in Switzerland

**DOI:** 10.1002/ajhb.23754

**Published:** 2022-04-30

**Authors:** Marc Rickenbacher, Nejla Gültekin, Zeno Stanga, Nicole Bender, Kaspar Staub, Jonathan C. Wells, Katarina L. Matthes, Emile Reber

**Affiliations:** ^1^ Department of Diabetes, Endocrinology, Nutritional Medicine and Metabolism University Hospital and University of Bern Bern Switzerland; ^2^ Institute of Evolutionary Medicine University of Zurich Zurich Switzerland; ^3^ Swiss Armed Forces, Medical Services Ittigen Switzerland; ^4^ Centre of Competence for Military and Disaster Medicine, Swiss Armed Forces Ittigen Switzerland; ^5^ Swiss School of Public Health SSPH+ Zurich Switzerland; ^6^ Zurich Center for Integrative Human Physiology (ZIHP), University of Zurich Zurich Switzerland; ^7^ Childhood Nutrition Research Centre, UCL Great Ormond Street Institute of Child Health London UK

## Abstract

**Object:**

Excess weight (Body Mass Index [BMI] ≥25.0 kg/m^2^) is a major health issue worldwide, including in Switzerland. For high‐income countries, little attention has been paid to body height in context of excess weight. The aim of this study is to assess the importance of body height as a co‐factor for excess weight in multiple large nationwide data sets.

**Data and methods:**

In this comparative study, we included the largest nationwide and population‐based studies in the fields of public health, nutrition and economics for Switzerland, as well as data of the medical examination during conscription for the Swiss Armed Forces, which contained information on BMI and, if possible, waist‐to‐height‐ratio (WHtR) and waist‐to‐hip‐ratio (WHR).

**Results:**

The multinomial logistic regressions show that the probability of belonging to the excess weight category (BMI ≥25.0 kg/m^2^) decreased with increasing height in both sexes inall contemporary data sets. This negative association was shown to be constant, only among conscripts measured in the 1870s the association was positive, when increasing height was associated with a higher BMI. The negative association not only emerge in BMI, but also in WHtR and WHR.

**Conclusion:**

Our results emphasize the importance of body height as a co‐factor of excess weight, suggesting a clear negative association between height and BMI, WHtR and WHR. Evidence indicates that both early‐life environmental exposures and alleles associated with height may contribute to these associations. This knowledge could serve as further starting points for prevention programs in the field of public health.

## 1 INTRODUCTION

Excess weight (Body Mass Index [BMI] ≥25.0 kg/m^2^) is a major health issue world‐wide, including in Switzerland, and therefore an important contributor to the global burden of chronic disease and disability for decades (Ng et al., 2014; World Health Organization (WHO), 2020). According to the Swiss Federal Office of Public Health, around 40% of the adult population and 15% of children and juveniles are currently suffering from excess weight (Bundesamt für Gesundheit (BAG), 2020). Excess weight and its associated comorbidities are responsible for a considerable share of the total healthcare expenses in Switzerland, representing an important economic and public health burden (Schneider & Venetz, 2014).

Excess weight is a highly multifactorial disease. Among contributing factors, lifestyle, especially the intake of energy‐dense food and/or decreased physical activity is of particular importance. Given that excess weight can be influenced by the factors mentioned above, prevention programs and other public health measures are playing a key role. However, for this to work, the determinants of excess weight must be understood as completely as possible.

To express and evaluate excess weight, BMI is the most frequently used anthropometric parameter. Its simple calculation and comparability are among the reasons for its wide usage, and at the population level, its correlation with body fat is fairly high (Keys et al., 1972; Kit et al., 2014). But besides population level, the applicability of BMI shows some clear limitations: especially short and tall people are often getting over‐ or underestimated, respectively, using BMI as a comparative reference. Additional, BMI does not allow to distinguish between fat and musculoskeletal tissue, consequently no statement can be made about health‐relevant body fat distribution (Burkhauser & Cawley, 2008; Henneberg & Veitch, 2005; H. J. Schneider et al., 2010). It is generally known, that body compositions differs significantly between the sexes, resulting in women having a higher percentage of body fat (Deurenberg et al., 1991; Gallagher et al., 1996; Roubenoff et al., 1995). A recent study also found out, that women tend to have a stronger negative association between BMI and body height (McGee, 2005; Sperrin et al., 2016).

Because of its limitations, other anthropometric variables are used to assess excess weight more precisely such as waist circumference (WC), waist‐to‐height‐ratio (WHtR), and waist‐to‐hip‐ratio (WHR). Compared to BMI, WC correlates more strongly with total body fat percentage and also allows conclusions to be drawn about body fat distribution (Higgins & Comuzzie, 2012; Lean et al., 1995; Malatesta, 2013; Singh, 2002). The WC maps in particular the visceral fat tissue, which is characteristic of central or abdominal obesity, which in turn is exceptionally strongly associated with diabetes mellitus type II or cardiovascular diseases (Clair et al., 2011; De Koning et al., 2007; Dis Ineke et al., 2009; Henneberg & Veitch, 2005; Lee et al., 2008; McCarthy & Ashwell, 2006; H. J. Schneider et al., 2010; Staiano et al., 2012; Wietlisbach et al., 2013; Xiao et al., 2006; Yusuf et al., 2005). For this reason, the WC is included in epidemiological studies on obesity and secondary diseases whenever possible. Since the WHO limits for WC are not applicable to particularly short or tall people, the use of the waist‐to‐height ratio (WHtR) has additionally been suggested in literature (Aeberli et al., 2011).

The discussion on how best to relate weight and height in humans in an index from a statistical perspective goes back to the 19th century and was extensively evaluated thereafter. One of the proposed solutions (to divide the weight by the square of the height) was proposed by Adolphe Quetelet and was supported subsequently by Ancel Keys as the so‐called Body Mass Index, becoming the backbone of health research and clinical practice in the modern era (Blackburn & Jacobs, 2014; Eknoyan, 2007; Keys et al., 1972). Nevertheless, among the biological co‐factors that contribute to obesity, body height has been understudied, especially in the public health field in high‐income countries. This is despite the fact that recent research has pointed out the importance of this factor.

This is not only shown by the increasingly important literature on the dual burden of malnutrition in middle‐ and low‐income countries, where populations face at the same time both high levels of stunting and excess weight (Black et al., 2013; Popkin et al., 2020). In addition, previous research on high income countries has shown a negative association between height and BMI. For 25 different populations from the U.S., Europe, and Asia it has been shown that body height is an important determinant of BMI (with a negative correlation found in 31 out of 40 subgroups in men and 32 of 32 subgroups in women), and that the relationship between weight and body height differs in men and women (McGee, 2005). Likewise, recent studies examined the correlation of height and BMI and found a similar relationship, proposing that being short is associated with having a higher BMI (Matthes et al., 2020; Staub et al., 2016). These correlations of BMI have already been mentioned in previous studies (Micozzi et al., 1986; Smalley et al., 1990).

It has been suggested, that the magnitude and sign of the correlation between height and BMI are age‐dependent (Johnson et al., 2020). It is positive in children and decreases with age, becoming negative in middle age (Garn et al., 1986). The underlying mechanisms are not yet fully clarified and are hypothesized to include variations in body proportions (taller people have longer leg lengths relative to shorter people, which affects body composition), some metabolic traits (e.g., later catch‐up growth in weight and BMI in children with previously poor growth in early life (Fagerberg et al., 2004; Lundgren et al., 2001), associations of alleles for greater height with lower BMI (Nüesch et al., 2016), and the documented non‐linear height‐weight relationship (Gunnell et al., 1998; Henneberg et al., 1989; Staub et al., 2016; Whitley et al., 2008). Altogether, environmental constraints in early life seem to contribute to the correlations of overweight and height in adult life. It is also not entirely clear to what extent confounding, for example through socio‐economic background, can play a role.

This underlines the importance of body height as a potential co‐factor of excess weight. But because body height was barely established and included as a co‐factor in discussions about excess weight, especially also in Switzerland, the goal of this comparative study was a) to assess the importance of height as a co‐factor across multiple large nationwide data sets, and b) to assess if this association changed across time.

## 2 DATA AND METHODS

In total, four representative national Swiss population‐based surveys and the data from the annual medical examination of Swiss conscripts were included in this study, an overview of all included datasets is shown in Supplementary Table S1.

### 2.1 Population based surveys

The *Swiss Household Panel (SHP)* is a representative longitudinal survey on social changes and living conditions of Swiss people and has been conducted every year since 1999 with three cohorts (1999, 2004 and 2013). Cross‐sectional data on the third sample population consisting of 4996 participants over the age of 18 years and obtained in 2014 were included in the statistical analysis. Self‐reported height and weight were obtained from all household members aged 15 years and over in individual telephone interviews. A more detailed description of the data collection, recruitment procedure, participant rate and sample weight strategy was published elsewhere (Voorpostel et al., 2020). *menuCH* is a representative Swiss national nutrition survey conducted between 2014 and 2015 collecting 24‐hours‐food‐intakte protocols. Survey data of 2036 participants between 18 and 75 years were included in the statistical analysis. Height and weight are available in both measured and self‐reported form. A more detailed description of the data collection, recruitment procedure and participation rate was published elsewhere (Bochud et al., 2017). The *Swiss Health Survey (SHS)* is a representative survey of health conditions, health behavior and the use of health services by people living in Switzerland and has been conducted every five years since 1992. Survey data from 2017including 20 946 participants over the age of 18 were includedin themain analysis. The previous 5 SHS since 1992 were also analyzed for a sensitivity analysis according to the same criteria. Self‐reported height and weight were obtained through structured telephone interviews. A more detailed description of the data collection, recruitment procedure, participant rate and sample weight strategy was published elsewhere (Bundesamt für Statistik, 2013; Storni et al., 2018). The *Swiss survey on Statistics on Income and Living Conditions (SILC)* is a representative study on income and living conditions of Swiss households. Households and their members are surveyed for several years, and new households are added each year. Cross‐sectional data on a total of 12 817 participants over the age of 18 obtained in 2017 were included in the statistical analysis. Self‐reported height and weight were obtained from household members aged 16 years and over in structured telephone interviews. A more detailed description of the data collection, recruitment procedure, participant rate and sample weight strategy was published elsewhere (Swiss Federal Statistical Office, 2014).

From all four surveys, we used participant self‐reported body height and body weight, in addition to that reported in the menuCH and conscripts, for which body height and body weight were measured, to calculate BMI (kg/m^2^). We included only participants with a BMI between 14.0 and 60.0 kg/m^2^. BMI was categorized using the WHO classification (World Health Organization, 2022): underweight (BMI <18.5 kg/m^2^), normal weight (18.5 BMI ‐ 24.9 kg/m^2^), and excess weight (BMI ≥25.0 kg/m^2^). In addition to BMI, waist and hip circumferences were measured and waist‐to‐hip‐ratio (WHR) and waist‐to‐height‐ratio (WHtR) were calculated in menuCH and grouped according to official categorisations: According to previous studies, participants were classified to be at risk for cardiovascular complications according to the following WHtR‐ and WHR cut‐offs or groups, for men (M) and women (W) respectively: WHR: (1) no risk <0.95 (M), < 0.80 (W), (2) increased risk 0.95–1.00 (M), 0.80–0.85 (W), (3) substantially increased risk >1.00 (M), > 0.85 (W); WHtR in both sexes: (1) no risk ≤0.5, (2) increased risk >0.5–0.6, (3) substantially increased risk >0.6 (Ashwell, 2011; World Health Organisation (WHO), 2008).

In all studies, information on sex, age, greater region (“Grossregion”), nationality, and education was available. The greater regions, were defined by the Swiss Federal Office of Statistics (Swiss Federal Statistical Office, 2019). Nationality was coded as either “Swiss” or “Non‐Swiss.” Residence status was available for 3 of the 4 studies (not in SILC), and was binary coded as either “urban” or “rural” (Swiss Federal Statistical Office, 2020).

In order to assess the socio‐economic background, we have chosen the variable “level of education,” which is included in all survey data sets according to the same categorization systems. Although some participants, especially in their younger years, have not yet completed their education, this variable has proven to be the most reliable in health research in Switzerland (Faeh et al., 2010). This is also because in Switzerland the distribution of the broad education categories is more equal (according to the Statistical Office, around 43% of the adult population had a tertiary education in 2017). In our SHS and SHP datasets, in addition to education level, we also have individual income as a variable (in SILC and menuCH, it is not individual income but household income, which gives a less precise picture). In SHS and SHP the agreement between income and education was very good (Supplementary Figure S1). For these reasons, we decided to rely on education as a proxy for socio‐economic background. Education was classified into three categories: primary (no degree or a compulsory school degree), secondary (completed high school or apprenticeship), and tertiary (higher degree for which a high school diploma was required).

All four data sets were made available by the Federal Food Safety and Veterinary Office (FSVO) and the Federal Statistical Office (FSO) in fully anonymized form, based on contractual agreement. Due to the anonymization of the data based on the Human Research Act (HRA) and before data delivery to the study team, there was no obligation to obtain approval from an ethics committee.

### 2.2 Conscription data

Another way of analyzing nationally representative anthropometric data is to use the health data of recruitment for the Swiss Armed Forces, which has been carried out in a standardized manner throughout the year since 1875. In the present study, we reuse data published previously in 2019 as well as from historical years (1990s, 1950s, 1930s, 1870s). These conscription data usually cover around 90–95% of all male Swiss citizens in early adulthood. The estimated 5–10% of male birth cohorts who do not show up for screening at conscription represent the full spectrum of severe physical and mental diseases and impairment, which are not necessarily associated with overweight and height (Panczak et al., 2014). Therefore, the coverage of the Swiss conscription data is considered very high and sample selection bias is not regarded as an issue.

The data from 2019 consist of 25 724 male conscripts between 18 and 21 years. In addition to the measurement of body height and weight, the Swiss Armed Forces have added waist circumference measurements as a new standard as of the beginning of 2019.

The occupation of the conscript is the only variable that gives an indication of the socio‐economic background at the individual level. According to previous studies, the occupations of the conscripts were assigned to the Socio‐Economic Index of Occupational Status (ISEI‐08). The ISEI allows the comparison of occupations according to their socio‐economic status. It can take on values between 16 (agricultural assistants) and 90 (judges) and was also used, for example, for the PISA studies as an indicator of socio‐economic status associated with occupation. The ISEI‐08 distribution of the occupations of those subject to compulsory military service was divided into three equally large groups (terciles). In addition, pupils, high school graduates and students together form a separate group, as well as those who have no or insufficient information on their occupation. The age of the conscripts was only available in one‐year groups, and the allocation to the major regions was based on the ZIP codes of the place of residence (assignment was done by Armed Forces personnel before data delivery).

The 1990s and 2019 data sets were made available by the Medical Service (Swiss Armed Forces ‐ Armed Forces Staff ‐ Medical Service Division) on a contractual basis in accordance with the legal data protection requirements. The data were exported from the Medical Information System of the Armed Forces (MEDISA) by the Medical Department and completely anonymized in accordance with the specifications before being handed over to the study team. Due to the anonymization of the data based on the Human Research Act (HRA) and before data delivery to the study team, there was no obligation to obtain approval from an ethics committee.

### 2.3 Statistical analyses

All analyses were carried out separately for men and women. The analysis was restricted to participants aged 18 years and over.

Multinomial logistic regression was used to predict probabilities of BMI, WHtR and WC in relation to body height. Except for the conscription data, sample weights were considered in the analysis. For menuCH, SHP, SHS, and SILC, probabilities are presented for median age, secondary education, Swiss nationality, and greater region “Zurich.” The probabilities of conscripts are shown for age = 19 years, “medium” education, and greater region “Zurich.” When comparing historical data on conscripts, probabilities are presented only for age = 19 years, since no information on education was available in 1990.

All statistical analyses were performed using R Version 4.0.5. The package “nnet” was used to perform the multinomial logistic regression models (Venables & Ripley, 2002) and ggplot2 (Wickham, 2009) to produce all figures.

## 3 RESULTS

In Table 1 we report the descriptive statistics and frequency distributions for all included data sets (the unweighted results are reported in Table 1). Mean heights in the population‐based survey datasets which cover the full age rangesspanned from 176.4 cm (SHP) to 177.5 cm (SHS2017 and SILC) in men (SDs 6.9–7.4 cm), and from 164.4 cm (menuCH) to 164.8 cm (SHP) cm in women (SDs 6.6–7.0). The conscripts, which only depict young men aged 18–22 years, were on average 178.7 cm tall in 2019 (SD = 6.6). The prevalence of excess weight (BMI ≥25.0 kg/m^2^) was similar across population‐based surveys, with men showing a prevalence between 50.3% and 56.1%, whereas the prevalence was markedly lower in women (range 29.5% to 34.4%). These marked sex differences are also evident in WHR and WHtR, where the prevalence of categories at higher risk is generally lower than for BMI, but still more frequent in men than in women. In the 2019 conscripts, the prevalence of excess weight was 26.3% as seen by BMI, and 17.5% as seen by WHtR. All population‐based studies show good agreement in terms of composition by age, nationality and larger regions. For education level, the category of tertiary education is generally slightly less frequent for women than for men, but in contrast it is higher for both sexes in menuCH.

**TABLE 1:** Descriptive data of the included data sets classified by sex, using the provided sample weights

Men							Women				
	SHP (*N* = 2 518 874)	menuCH (*N* = 2 268 762)	SHS2017 (*N* = 3 315 452)	SILC (*N* = 3 324 765)		Conscripts^a^ (*N* = 25 724)		SHP (*N* = 2 463 336)	menuCH (*N* = 2 250 695)	SHS2017 (*N* = 3 389 948)	SILC (*N* = 3 378 841)
*Height*					*Height*		*Height*				
Mean (SD)	177.1 (7.0)	176.4 (7.4)	177.5 (7.2)	177.5 (6.9)	Mean (SD)	178.7 (6.6)	Mean (SD)	164.8 (6.6)	164.4 (7.0)	164.7 (6.6)	164.7 (6.6)
Range	143.0–202.0	153.5–202.0	142.0–210.0	143.0–206.0	Range	150.0–210.0	Range	141.0–188.0	139.6–189.9	136.0–193.0	135.0–195.0
*BMIgroup*					*BMIgroup*		*BMIgroup*				
<18.5	15 902 (0.6%)	17 340 (0.8%)	27 013 (0.8%)	25 832 (0.8%)	< 18.5	1329 (5.2%)	< 18.5	131 351 (5.3%)	87 213 (3.9%)	164 834 (4.9%)	155 373 (4.6%)
18.5–24.9	1 236 537 (49.1%)	978 570 (43.1%)	1 544 593 (46.6%)	1 522 797 (45.8%)	18.5–24.9	17 622 (68.5%)	18.5–24.9	1 527 720 (62.0%)	1 500 293 (66.7%)	2 060 232 (60.8%)	2 060 570 (61.0%)
≥ 25.0	1 266 435 (50.3%)	1 272 851 (56.1%)	1 743 846 (52.6%)	1 776 137 (53.4%)	≥25.0	6773 (26.3%)	≥25.0	804 265 (32.6%)	663 188 (29.5%)	1 164 883 (34.4%)	1 162 898 (34.4%)
*WHRgroup*					*WHRgroup*		*WHRgroup*				
<0.95	‐	1 313 701 (57.9%)	‐	‐	<0.95	‐	<0.8	‐	1 639 074 (72.8%)	‐	‐
0.95–1.0	‐	664 513 (29.3%)	‐	‐	0.95–1.0	‐	0.8–0.85	‐	286 623 (12.7%)	‐	‐
>1.0	‐	290 548 (12–8%)	‐	‐	>1.0	‐	>0.85	‐	324 998 (14.4%)	‐	‐
*WHtRgroup*					*WtHRgroup*		*WtHRgroup*				
≤0.5	‐	1 099 763 (48.5%)	‐	‐	≤0.5	20 458 (79.5%)	≤0.5	‐	1 658 214 (73.7%)	‐	‐
>0.5–0.6	‐	906 206 (39.9%)	‐	‐	>0.5–0.6	3795 (14.8%)	> 0.5–0.6	‐	440 456 (19.6%)	‐	‐
>0.6	‐	262 793 (11.6%)	‐	‐	>0.6	687 (2.7%)	>0.6	‐	152 024 (6.8%)	‐	‐
					Missing	784 (3.0%)					
*Education*					*Education*		*Education*				
Primary	264 967 (10.5%)	113 635 (5.0%)	423 460 (12.8%)	411 280 (12.4%)	Low	5032 (19.6%)	Primary	332 436 (13.5%)	96 453 (4.3%)	628 577 (18.5%)	615 395 (18.2%)
Secondary	1 312 210 (52.1%)	898 765 (39.6%)	1 527 996 (46.1%)	1 659 470 (49.9%)	Medium	6623 (25.7%)	Secondary	1 449 359 (58.8%)	1 043 106 (46.3%)	1 771 386 (52.3%)	1 765 520 (52.3%)
Tertiary	941 697 (37.4%)	1 256 361 (55.4%)	1 363 997 (41.1%)	1 254 016 (37.7%)	High	4183 (16.3%)	Tertiary	681 541 (27.7%)	1 111 136 (49.4%)	989 985 (29.2%)	997 927 (29.5%)
					Students	5894 (22.9%)					
					Imprecise	3992 (15.5%)					
*Age*					*Age groups*		*Age*				
Mean (SD)	47.4 (17.4)	46.3 (15.6)	48.0 (17.7)	48.0 (17.4)	<19.00	8788 (34.2%)	Mean (SD)	48.9 (18.3)	45.1 (15.2)	49.2 (18.4)	48.9 (17.8)
Range	18.0–95.0	18.0–76.0	18.0–98.0	18.0–80.0	≥22.00	1333 (5.2%)	Range	18.0–97.0	18.0–76.0	18.0–103.0	18.0–80.0
					19.00–19.99	10 145 (39.4%)					
					20.00–20.99	4287 (16.7%)					
					21.00–21.99	1171 (4.6%)					
*Region*					*Region*		*Region*				
Espace Mittelland	537 337 (21.3%)	462 789 (20.4%)	708 354 (21.4%)	725 902 (21.8%)	Espace Mittelland	6279 (24.4%)	Espace Mittelland	524 878 (21.3%)	449 461 (20.0%)	776 496 (22.9%)	755 907 (22.4%)
Genferseeregion	451 793 (17.9%)	390 562 (17.2%)	605 641 (18.3%)	604 741 (18.2%)	Genferseeregion	3882 (15.1%)	Genferseeregion	475 142 (19.3%)	473 660 (21.0%)	633 924 (18.7%)	653 846 (19.4%)
Nordwestschweiz	356 261 (14.1%)	408 913 (18.0%)	443 698 (13.4%)	467 127 (14.0%)	Nordwestschweiz	3458 (13.4%)	Nordwestschweiz	331 894 (13.5%)	375 404 (16.7%)	481 269 (14.2%)	449 070 (13.3%)
Ostschweiz	381 325 (15.1%)	228 406 (10.1%)	479 413 (14.5%)	482 774 (14.5%)	Ostschweiz	4037 (15.7%)	Ostschweiz	354 318 (14.4%)	132 453 (5.9%)	453 079 (13.4%)	456 627 (13.5%)
Tessin	133 520 (5.3%)	125 414 (5.5%)	143 875 (4.3%)	137 680 (4.1%)	Tessin	1082 (4.2%)	Tessin	111 707 (4.5%)	126 920 (5.6%)	143 678 (4.2%)	151 422 (4.5%)
Zentralschweiz	220 162 (8.7%)	146 720 (6.5%)	333 232 (10.1%)	321 819 (9.7%)	Zentralschweiz	2831 (11.0%)	Zentralschweiz	208 936 (8.5%)	133 686 (5.9%)	306 010 (9.0%)	318 708 (9.4%)
Zurich	438 476 (17.4%)	505 958 (22.3%)	601 239 (18.1%)	584 721 (17.6%)	Zurich	4155 (16.2%)	Zurich	456 461 (18.5%)	559 111 (24.8%)	595 492 (17.6%)	593 261 (17.6%)
*Nationality*					*Nationality*		*Nationality*				
Non‐Swiss	640 055 (25.4%)	627 334 (27.7%)	872 265 (26.3%)	886 493 (26.7%)	Non‐Swiss	‐	Non‐Swiss	581 653 (23.6%)	616 821 (27.4%)	758 293 (22.4%)	785 886 (23.3%)
Swiss	1 878 819 (74.6%)	1 641 427 (72.3%)	2 443 187 (73.7%)	2 438 272 (73.3%)	Swiss	25 724 (100.0%)	Swiss	1 881 683 (76.4%)	1 633 874 (72.6%)	2 631 656 (77.6%)	2 592 955 (76.7%)

*Abbreviations*: BMI, body mass index; SHP, Swiss household panel; SHS, Swiss health survey; SILC, survey on statistics on income and living conditions; WHR, waist‐to‐hip‐ratio; WHtR, weight‐to‐height‐ratio.

^a^ Unweighted data.

**FIGURE 1:**
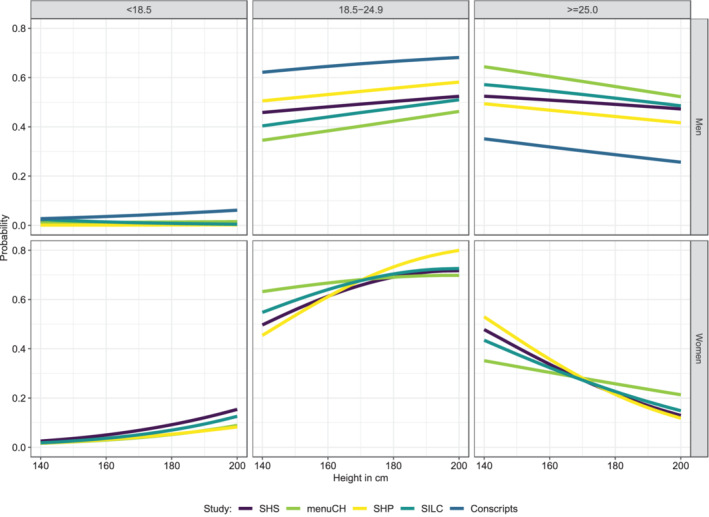
Probabilities of belonging to one of the three BMI groups as a function of height in all included data sets. For menuCH, SHP, SHS, and SILC, probabilities are presented for median age, secondary education, Swiss nationality, and greater region “Zurich.” The probabilities of conscripts are shown for age = 19 years, “medium” education, and greater region “Zurich.” (SHP = Swiss Houshold panel, SHS = Swiss Health Survey, SILC = Survey on Statistics on Income and Living Conditions).

**FIGURE 2:**
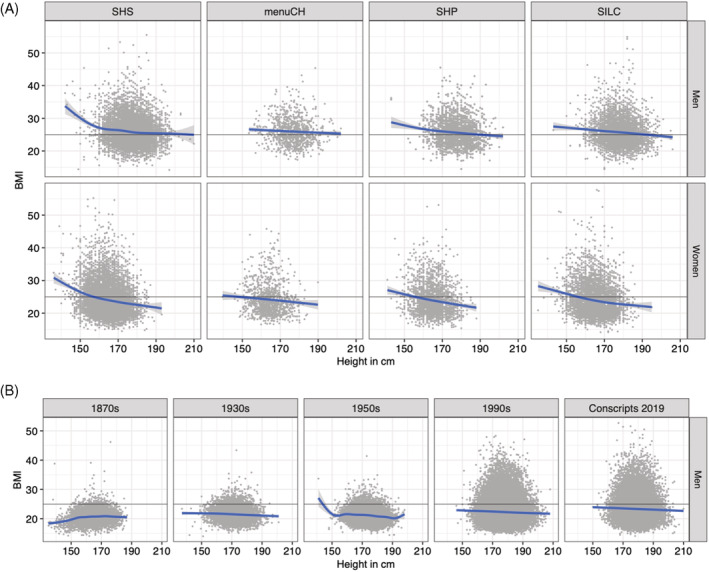
Scatterplots of all datasets (A: Population based surveys; B: Conscripts across time) stratified by sex showing the negative association between height and BMI. The blue lines indicate smoothed trends using a gam function.

The multinomial logistic regressions show that the probability of belonging to the excess weight category (BMI ≥25.0 kg/m^2^) decreased with increasing height in both sexes and all contemporary data sets (SHP, SHS2017, menuCH, SILC, conscripts) (Figure 1). This pattern was even more pronounced in women than in men. In contrast, the probability belonging to the normal‐weight category (BMI 18.5–24.9 kg/m^2^) increased with increasing height for both sexes, and the same was apparent in the under‐weight category (BMI < 18.5 kg/m^2^) for women. A sensitivity analysis comparing unadjusted vs. adjusted effects in SHS 2017 reveals that the effects are still apparent in the adjusted models (Supplementary Figure S2). Regarding co‐factors in the regressions, the following patterns emerged relatively consistently: the older the person, the higher the BMI, while in addition, higher educated people and those with Swiss nationality had lower BMI. The negative association between height and BMI in each of the data sets is also shown as scatterplots in Figure 2. A sensitivity analysis, which additionally included the urban/rural variable in 4 of the 5 data sets, showed very similar results and did not change the general conclusion (results not shown).

**FIGURE 3:**
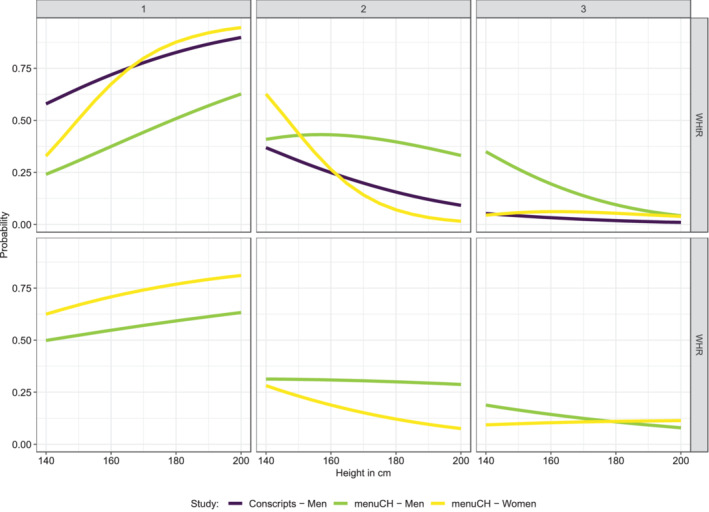
Probabilities of belonging to one of the threeWHtR‐ and WHR‐groups as a function of heightin the datasets menuCH and conscripts. For menuCH probabilities are presented for median age, secondary education, Swiss nationality, and greater region “Zurich.” The probabilities of conscripts are shown for age = 19 years, “medium” education, and greater region “Zurich.” WHtR was classified into 1:≤0.5, 2:>0.5–0.6, 3:>0.6. WHRwas categorized into:1:< 0.95 (men), < 0.8 (women), 2: 0.95–1.0 (men), 0.8–0.85 (women), 3 > 1.0 (men), > 0.85 (women).

**FIGURE 4:**
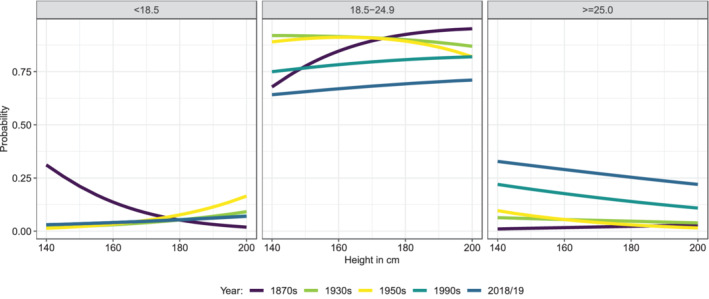
Comparison of the probabilities to belong to the BMI‐groups among conscriptsmeasured in the 1870s, 1930s, 1950s, 1990s, and 2018/2019. The probabilities are shown for median age and “medium” education.

If we run the same models on the menuCH and conscriptiondata that had also information on WHtR and WHR, we see that the same pattern emerges for these other anthropometric measures: the taller the individual, the higher the probability of belonging to the normal‐weighted category (Figure 3). In asking whether the negative association between body height and BMI has changed over time, we can draw on all other five SHS since 1992 on the one hand, and on conscription data since the 1870s on the other. Comparing all SHS (Supplementary Figure S3) shows that the pattern is very similar and stable across all SHS since 1992. In the case of the conscripts, it becomes apparent that only the 1870s conscription years behave in the opposite way (Figure 4): Whereas excess weight was still rare at that time, the probability of belonging to the underweight category (BMI < 18.5 kg/m^2^) decreases with increasing height. In the 1870s, the conscripts were only 164.8 cm tall by average, and the association of height with BMI was positive, and only in the 20th century the association became negative.

## 4 DISCUSSION

We present the first comparative analysis on the height‐BMI relationship in Switzerland based on various representative population‐wide datasets. The common pattern emerging from all included contemporary data sets is that the probability to belong to the excess weight group decreases with increasing body height. Height and BMI were negatively associated (the taller the study participant, the lower the BMI), even after adjusting for co‐factors like age, education level, nationality, and region. In women, the pattern was stronger than in men. When looking at changes of this height‐BMI association across time in the SHS and conscript data sets, the pattern emerges constantly across the 20th century (the association was positive in conscripts only at the end of the 19th century). Most importantly, a negative association between height and excess weight was also found when using WHtR and WHR (instead of BMI) as dependent variables (and proxies for abdominal and hip fat): The taller the individual, the higher the probability to belong to both the normal‐weight / no‐risk categories, and for the women, an increased risk of being in the underweight BMI category. In summary, the negative association between height and excess weight seems to be a general pattern, at least in Switzerland, for both sexes and all age groups. This negative association tends to be even more pronounced in women than in men, supporting results of previous research (McGee, 2005; Sperrin et al., 2016). A possible explanation for this phenomenon might be the different conditions at a young age, since greater maternal pregnancy weight gain (Fraser et al., 2010), higher birth weight (Yu et al., 2011) and faster growth (Buchan et al., 2007; Metcalf et al., 2011) are associated with increased BMI at puberty, which in turn is again associated with increased BMI at adolescence (De Leonibus et al., 2014; Zheng et al., 2014).

The literature on excess weight in Switzerland has so far overlooked height as important co‐factor, hence this study is the first to point in this direction. We confirm, however, other studies which also show this association across data sets and adult populations (Garn et al., 1986; Matthes et al., 2020; Micozzi et al., 1986; Smalley et al., 1990; Staub et al., 2016). Future studies in Switzerland should thus also look at the role of height on excess weight in children, even though the analysis and interpretation of height, weight and BMI in childhood is more challenging because of the ongoing dynamic change process of physical growth.

The mechanisms behind the negative association between body height and BMI shown here are not entirely clear at the moment, and may involve statistical, epidemiological, biological and genetic issues. From a statistical perspective, it has already been shown that the relationship between body height and body weight is not linear, and thus that BMI can be distorted in particularly short and tall people. However, we show here in this study that the negative association with height applies not only to BMI but also to WHtR and WHR, and thus the phenomenon is more complex. From an epidemiological perspective, the association of height and BMI might be confounded by other variables. While we adjust the models for education levels (which in public health research for Switzerland is the most reliable single indicator for socio‐economic position (Faeh et al., 2010), it is possible that residual confounding by socioeconomic background still plays some role. However, the common findings across cohorts here, and across populations in the literature, makes such confounding unlikely.

From a biological level, aspects of the development of body proportions and metabolic traits may contribute. For example, different body proportions in shorter and taller people may play some role: taller people have longer leg lengths relative to shorter people, which affects body composition. Of relevance here, leg length is particularly susceptible to nutritional constraints in early life (Gunnell et al., 1998; Whitley et al., 2008). Moreover, poor growth in early life has been linked in numerous studies with subsequent catch‐up in weight and BMI, potentially mediated by alterations in appetite (Barker et al., 2005). Analysis of a large database of 3D body shape found that whereas most body girths were positively associated with height, waist girth showed a strong inverse association in men, while waist, thigh and arm girths showed weaker inverse associations in women (Wells et al., 2007). Other studies have reported associations between short stature, impaired fat oxidation central body fatness in Brazilian children (Hoffman et al., 2000) and Siberian women (Leonard et al., 2009). Collectively, these studies suggest that environmental constraints in early life may contribute to correlations of overweight and height in adult life. We were unable to investigate this issue in our studies because we did not have data on sitting height or leg length available for any cohort. Finally, genetic mechanisms may also contribute. A Mendelian randomization study reported a causal association of tall height with both lower BMI and lower non‐communicable disease risk (Nüesch et al., 2016). This suggests that the causal benefits of taller height for adult health may be partly mediated by lower BMI.

That the relation between body height and BMI was first positive at the end of the 19th century (taller height = higher BMI), and then became negative in the course of the 20th century, has already been shown elsewhere and discussed in detail (Staub et al., 2016). That particularly short people also tended to be thinner in the late 19th century reflects a double burden of the lower socioeconomic groups in prevailing conditions of undernutrition (12% of the conscripts were underweight by modern WHO‐standards) and food uncertainty. The rising standard of living in Switzerland around 1900 had the effect of changing these conditions, which is reflected in the reversal of the height to BMI relationship. Today, in a situation of a negative height to BMI relation (shorter height = higher BMI), this double burden still exists, but differently. A rather unhealthy lifestyle during the growing years in childhood possibly leads to overweight already at younger age and to shorter adult height. The same lifestyle in adulthood exacerbates the overweight.

Our study has strengths and weaknesses. One strength is that, to our knowledge, this is the first comparative study of the height vs. overweight relationship that brings together and compares several large and representative data within a single country. This contributes to the robustness and generalizability of our results. An additional strength is that the included surveys are from different years and used different methods for sampling and data collection, suggesting that the results are not an artifact of a particular sampling approach. The included studies were all collected at different times between 2010 and 2019 and thus influences of temporal change (trends) would be possible. However, this possible limitation is put into perspective by the fact that, on the one hand, the monitoring datasets of schoolchildren and conscripts have shown a stable trend since 2010 and, on the other hand, there was no further significant increase in overweight and obesity between the two Swiss Health Surveys in 2012 and 2017. However, remaining differences between the data sets cannot be excluded. Another limitation, which we have already described in the data description, is that we could only use the level of education as an indicator of socioeconomic background in the surveys, although this was available in comparable form in all included data sets. Especially for participants at a younger age, the education level may still be incomplete. Other aspects of socioeconomic background (e.g., personal income) were only present in some of the data sets. However, since the correlation between income and education was strong in these data sets, and education has proven to be the most reliable socioeconomic variable in the Swiss public health research context (Faeh et al., 2010), we think that our results at least reflect the right tendency. The most important limitation is certainly that the anthropometric measures in most of the included population‐based survey datasets were not measured, but asked for via questionnaires and in interviews. This can lead to a self‐reporting bias (Bopp & Faeh, 2008; Vinci et al., 2019). However, all other data sets based on measured data point in the same direction, which gives our results overall credibility.

## 5 CONCLUSIONS

Our findings suggest that in the public health field and obesity discourse, too little emphasis has been placed on the body height variable in the BMI equation. The importance of the height variable should be recognized because it opens a life course dimension (Bundesamt für Gesundheit (BAG), 2016) and the overall role of development in the height vs. excess weight association needs to be better understood, also in terms of underlying mechanisms.

## CONFLICT OF INTEREST

The authors do not declare a conflict of interest.

## Supporting information


**Appendix S1** Supporting Information

## Data Availability

This article reuses datasets from various Swiss authorities (Swiss Armed Forces, Federal Food Safety and Veterinary Office, the Federal Statistical Office), which can be obtained through these authorities (via submitting a study protocol and signing data contracts). The authors of the article themselves are not authorized by contract to make individual datasets publicly available. The R‐codes for the analysis of the datasets can be obtained from the corresponding author.
