# Supplementary material for: The role of body height as a co‐factor of excess weight in Switzerland
Source: Am J Hum Biol. 2022 Apr 30;34(8):e23754. doi: 10.1002/ajhb.23754 (PMC9541525; doi:10.1002/ajhb.23754)
Supplement: Supplementary file 1 — Appendix S1 Supporting Information [file AJHB-34-e23754-s001.docx]

**Supplementary Material**

**
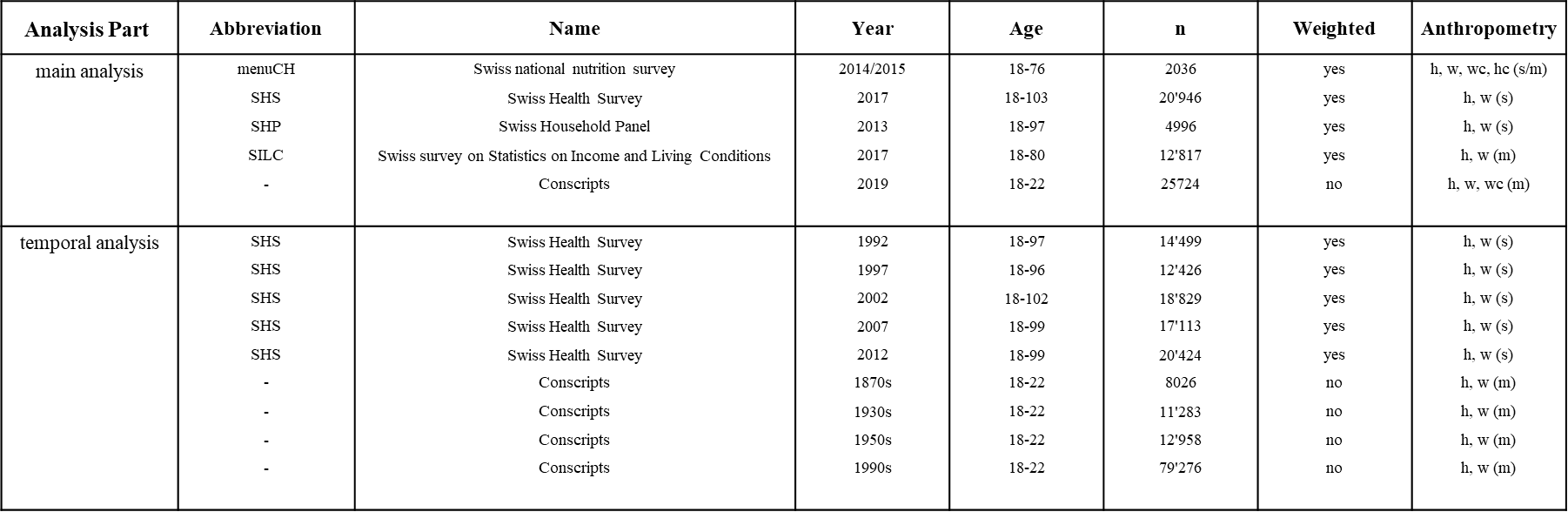
Supplementary Table S1:** Overview of included data sets (h = height, w = weight, wc = waist circumference, hc = hip circumference, s = self-reported, m = measured)

**Supplementary Figure S1:**Agreement between personal income (in Swiss Francs) and education level in the SHS and SHP datasets as displayed in boxplots.

******

**Supplementary Figure S2:** Comparison adjusted vs. unadjusted probabilities to belong to BMI-groups in the SHS 2017, stratified by sex.Probabilities are presented for median age, secondary education, Swiss nationality, and greater region “Zurich”.

**Supplementary Figure S3:** Comparing the probabilities to belong to WHO-groups for BMI across all six SHS since 1992. Probabilities are presented for median age, secondary education, Swiss nationality, and greater region “Zurich”.
